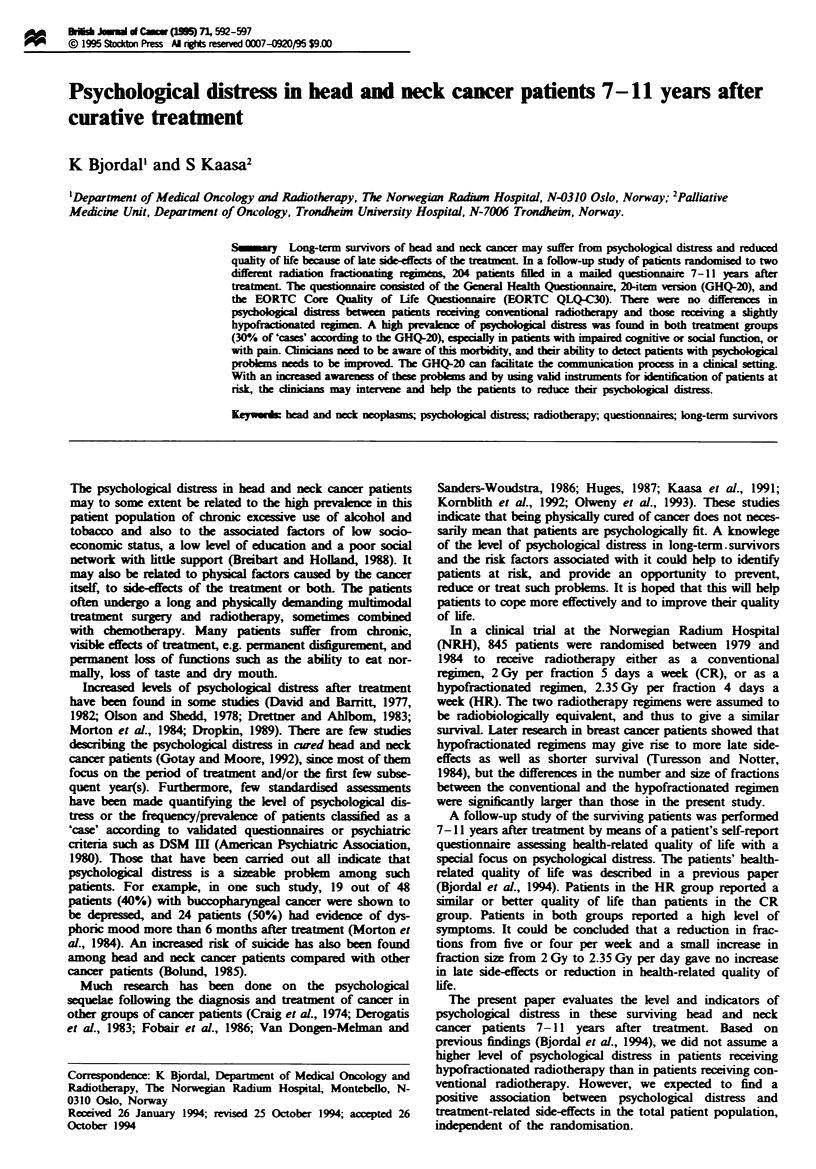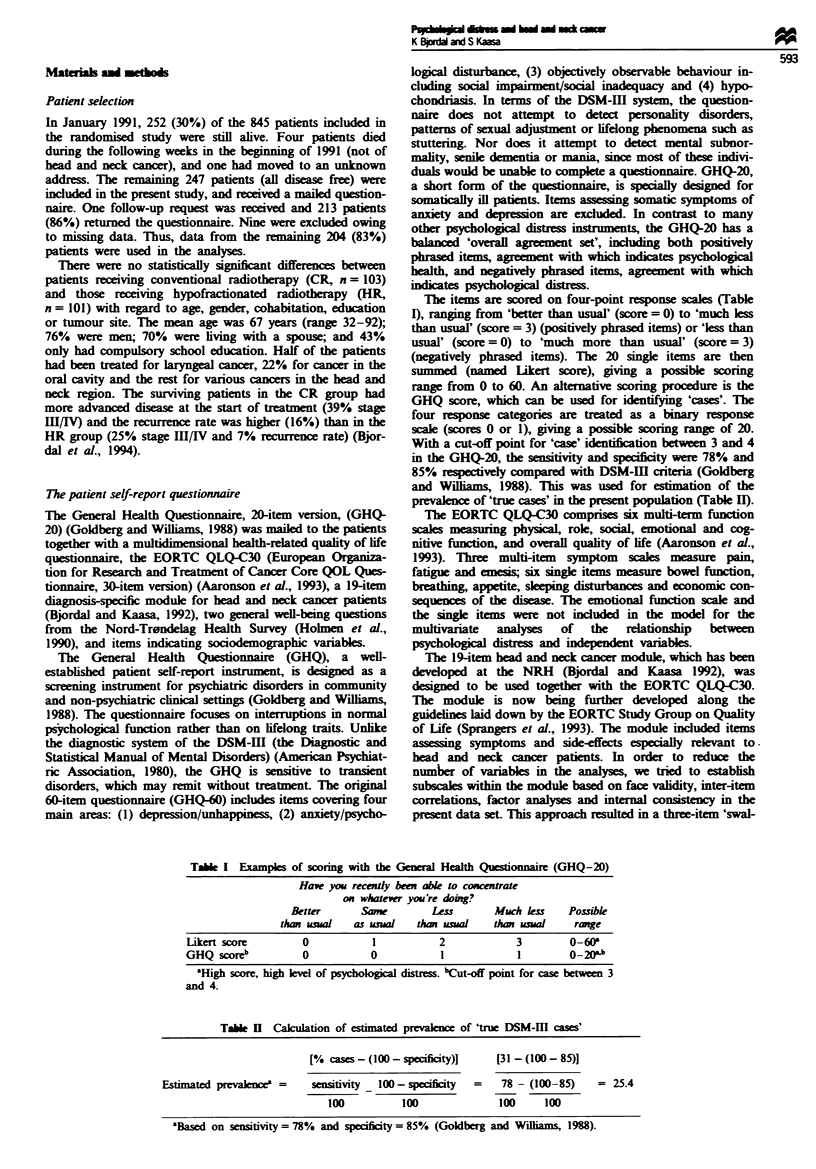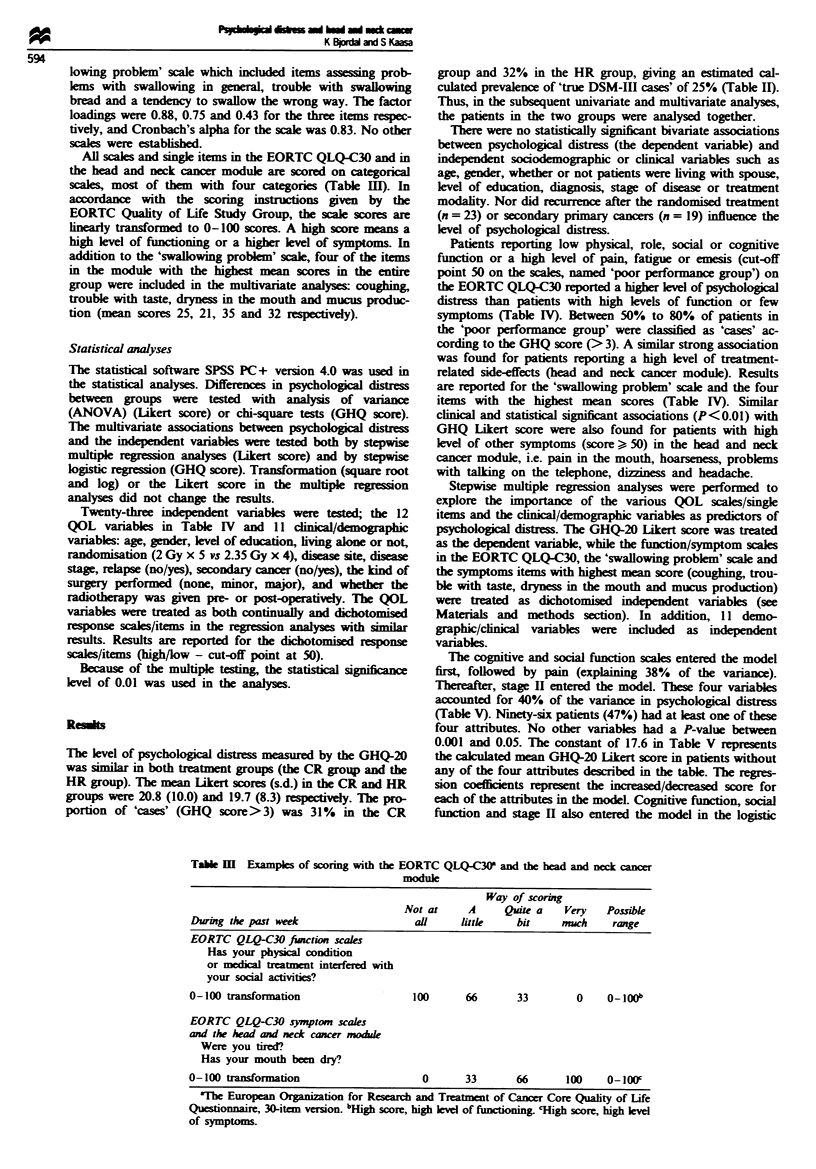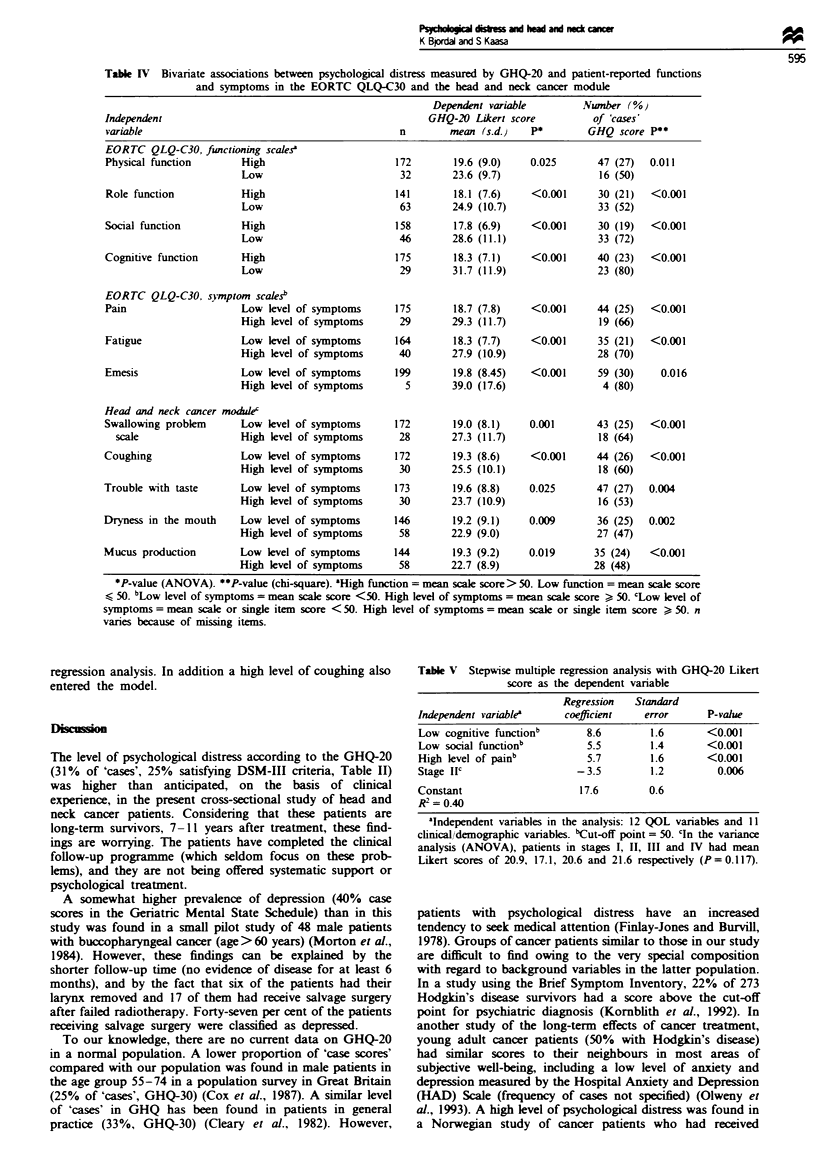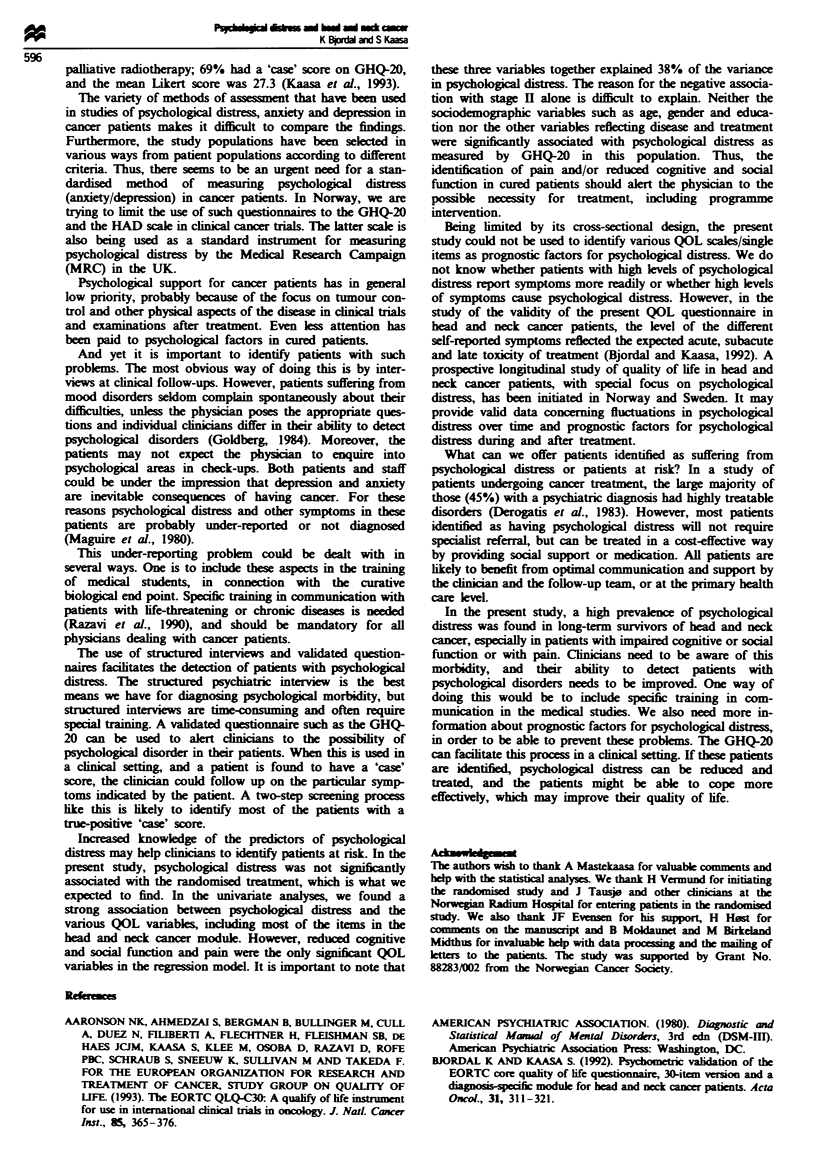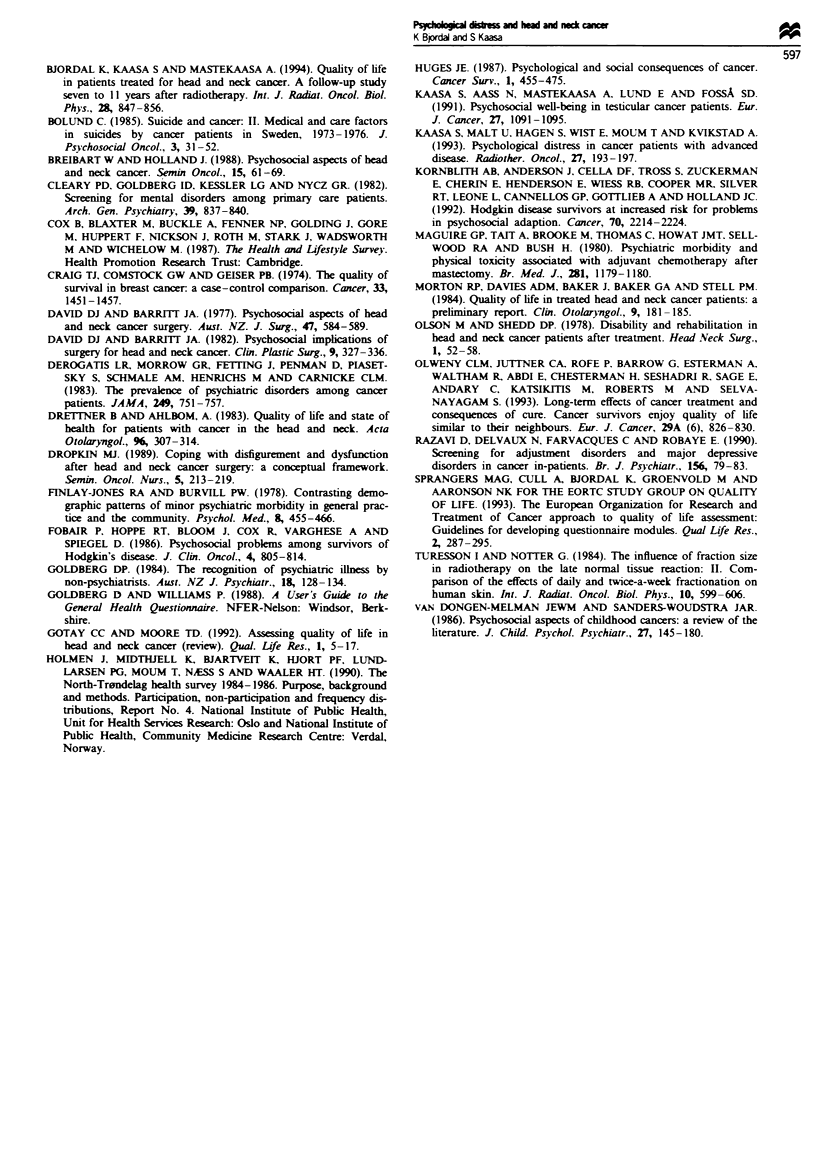# Psychological distress in head and neck cancer patients 7-11 years after curative treatment.

**DOI:** 10.1038/bjc.1995.115

**Published:** 1995-03

**Authors:** K. Bjordal, S. Kaasa

**Affiliations:** Department of Medical Oncology and Radiotherapy, Norwegian Radium Hospital, Oslo.

## Abstract

Long-term survivors of head and neck cancer may suffer from psychological distress and reduced quality of life because of late side-effects of the treatment. In a follow-up study of patients randomised to two different radiation fractionating regimens, 204 patients filled in a mailed questionnaire 7-11 years after treatment. The questionnaire consisted of the General Health Questionnaire, 20-item version (GHQ-20), and the EORTC Core Quality of Life Questionnaire (EORTC QLQ-C30). There were no differences in psychological distress between patients receiving conventional radiotherapy and those receiving a slightly hypofractionated regimen. A high prevalence of psychological distress was found in both treatment groups (30% of 'cases' according to the GHQ-20), especially in patients with impaired cognitive or social function, or with pain. Clinicians need to be aware of this morbidity, and their ability to detect patients with psychological problems needs to be improved. The GHQ-20 can facilitate the communication process in a clinical setting. With an increased awareness of these problems and by using valid instruments for identification of patients at risk, the clinicians may intervene and help the patients to reduce their psychological distress.


### Psychological distress in head and neck cancer patients 7-11 years after curative treatment

K Bjordal[1] and S Kaasa[2]

[1]*Department of Medical Oncology and Radiotherapy, The Norwegian Radium Hospital, N-0310 Oslo, Norway;* [2]*Palliative Medicine Unit, Department of Oncology, Trondheim University Hospital, N-7006 Trondheim, Norway.*

**Summary** Long-term survivors of head and neck cancer may suffer from psychological distress and reduced quality of life because of late side-effects of the treatment. In a follow-up study of patients randomised to two different radiation fractionating regimens, 204 patients filled in a mailed questionnaire 7-11 years after treatment. The questionnaire consisted of the General Health Questionnaire, 20-item version (GHQ-20), and the EORTC Core Quality of Life Questionnaire (EORTC QLQ-C30). There were no differences in psychological distress between patients receiving conventional radiotherapy and those receiving a slightly hypofractionated regimen. A high prevalence of psychological distress was found in both treatment groups (30% of 'cases' according to the GHQ-20), especially in patients with impaired cognitive or social function, or with pain. Clinicians need to be aware of this morbidity, and their ability to detect patients with psychological problems needs to be improved. The GHQ-20 can facilitate the communication process in a clinical setting. With an increased awareness of these problems and by using valid instruments for identification of patients at risk, the clinicians may intervene and help the patients to reduce their psychological distress.

**Keywords:** head and neck neoplasms; psychological distress; radiotherapy; questionnaires; long-term survivors

The psychological distress in head and neck cancer patients may to some extent be related to the high prevalence in this patient population of chronic excessive use of alcohol and tobacco and also to the associated factors of low socio-economic status, a low level of education and a poor social network with little support (Breibart and Holland, 1988). It may also be related to physical factors caused by the cancer itself, to side-effects of the treatment or both. The patients often undergo a long and physically demanding multimodal treatment surgery and radiotherapy, sometimes combined with chemotherapy. Many patients suffer from chronic, visible effects of treatment, e.g. permanent disfigurement, and permanent loss of functions such as the ability to eat normally, loss of taste and dry mouth.

Increased levels of psychological distress after treatment have been found in some studies (David and Barritt, 1977, 1982; Olson and Shedd, 1978; Drettner and Ahlbom, 1983; Morton *et al.*, 1984; Dropkin, 1989). There are few studies describing the psychological distress in *cured* head and neck cancer patients (Gotay and Moore, 1992), since most of them focus on the period of treatment and/or the first few subsequent year(s). Furthermore, few standardised assessments have been made quantifying the level of psychological distress or the frequency/prevalence of patients classified as a 'case' according to validated questionnaires or psychiatric criteria such as DSM III (American Psychiatric Association, 1980). Those that have been carried out all indicate that psychological distress is a sizeable problem among such patients. For example, in one such study, 19 out of 48 patients (40%) with buccopharyngeal cancer were shown to be depressed, and 24 patients (50%) had evidence of dysphoric mood more than 6 months after treatment (Morton *et al.*, 1984). An increased risk of suicide has also been found among head and neck cancer patients compared with other cancer patients (Bolund, 1985).

Much research has been done on the psychological sequelae following the diagnosis and treatment of cancer in other groups of cancer patients (Craig *et al.*, 1974; Derogatis *et al.*, 1983; Fobair *et al.*, 1986; Van Dongen-Melman and Sanders-Woudstra, 1986; Huges, 1987; Kaasa *et al.*, 1991; Kornblith *et al.*, 1992; Olweny *et al.*, 1993). These studies indicate that being physically cured of cancer does not necessarily mean that patients are psychologically fit. A knowlege of the level of psychological distress in long-term survivors and the risk factors associated with it could help to identify patients at risk, and provide an opportunity to prevent, reduce or treat such problems. It is hoped that this will help patients to cope more effectively and to improve their quality of life.

In a clinical trial at the Norwegian Radium Hospital (NRH), 845 patients were randomised between 1979 and 1984 to receive radiotherapy either as a conventional regimen, 2 Gy per fraction 5 days a week (CR), or as a hypofractionated regimen, 2.35 Gy per fraction 4 days a week (HR). The two radiotherapy regimens were assumed to be radiobiologically equivalent, and thus to give a similar survival. Later research in breast cancer patients showed that hypofractionated regimens may give rise to more late side-effects as well as shorter survival (Turesson and Notter, 1984), but the differences in the number and size of fractions between the conventional and the hypofractionated regimen were significantly larger than those in the present study.

A follow-up study of the surviving patients was performed 7-11 years after treatment by means of a patient's self-report questionnaire assessing health-related quality of life with a special focus on psychological distress. The patients' health-related quality of life was described in a previous paper (Bjordal *et al.*, 1994). Patients in the HR group reported a similar or better quality of life than patients in the CR group. Patients in both groups reported a high level of symptoms. It could be concluded that a reduction in fractions from five or four per week and a small increase in fraction size from 2 Gy to 2.35 Gy per day gave no increase in late side-effects or reduction in health-related quality of life.

The present paper evaluates the level and indicators of psychological distress in these surviving head and neck cancer patients 7-11 years after treatment. Based on previous findings (Bjordal *et al.*, 1994), we did not assume a higher level of psychological distress in patients receiving hypofractionated radiotherapy than in patients receiving conventional radiotherapy. However, we expected to find a positive association between psychological distress and treatment-related side-effects in the total patient population, independent of the randomisation.

Correspondence: K Bjordal, Department of Medical Oncology and Radiotherapy, The Norwegian Radium Hospital, Montebello, N-0310 Oslo, Norway
Received 26 January 1994; revised 25 October 1994; accepted 26 October 1994

### Materials and methods

### *Patient selection*

In January 1991, 252 (30%) of the 845 patients included in the randomised study were still alive. Four patients died during the following weeks in the beginning of 1991 (not of head and neck cancer), and one had moved to an unknown address. The remaining 247 patients (all disease free) were included in the present study, and received a mailed questionnaire. One follow-up request was received and 213 patients (86%) returned the questionnaire. Nine were excluded owing to missing data. Thus, data from the remaining 204 (83%) patients were used in the analyses.

There were no statistically significant differences between patients receiving conventional radiotherapy (CR, $n = 103$) and those receiving hypofractionated radiotherapy (HR, $n = 101$) with regard to age, gender, cohabitation, education or tumour site. The mean age was 67 years (range 32–92); 76% were men; 70% were living with a spouse; and 43% only had compulsory school education. Half of the patients had been treated for laryngeal cancer, 22% for cancer in the oral cavity and the rest for various cancers in the head and neck region. The surviving patients in the CR group had more advanced disease at the start of treatment (39% stage III/IV) and the recurrence rate was higher (16%) than in the HR group (25% stage III/IV and 7% recurrence rate) (Bjordal *et al.*, 1994).

### *The patient self-report questionnaire*

The General Health Questionnaire, 20-item version, (GHQ-20) (Goldberg and Williams, 1988) was mailed to the patients together with a multidimensional health-related quality of life questionnaire, the EORTC QLQ-C30 (European Organization for Research and Treatment of Cancer Core QOL Questionnaire, 30-item version) (Aaronson *et al.*, 1993), a 19-item diagnosis-specific module for head and neck cancer patients (Bjordal and Kaasa, 1992), two general well-being questions from the Nord-Trøndelag Health Survey (Holmen *et al.*, 1990), and items indicating sociodemographic variables.

The General Health Questionnaire (GHQ), a well-established patient self-report instrument, is designed as a screening instrument for psychiatric disorders in community and non-psychiatric clinical settings (Goldberg and Williams, 1988). The questionnaire focuses on interruptions in normal psychological function rather than on lifelong traits. Unlike the diagnostic system of the DSM-III (the Diagnostic and Statistical Manual of Mental Disorders) (American Psychiatric Association, 1980), the GHQ is sensitive to transient disorders, which may remit without treatment. The original 60-item questionnaire (GHQ-60) includes items covering four main areas: (1) depression/unhappiness, (2) anxiety/psychological disturbance, (3) objectively observable behaviour including social impairment/social inadequacy and (4) hypochondriasis. In terms of the DSM-III system, the questionnaire does not attempt to detect personality disorders, patterns of sexual adjustment or lifelong phenomena such as stuttering. Nor does it attempt to detect mental subnormality, senile dementia or mania, since most of these individuals would be unable to complete a questionnaire. GHQ-20, a short form of the questionnaire, is specially designed for somatically ill patients. Items assessing somatic symptoms of anxiety and depression are excluded. In contrast to many other psychological distress instruments, the GHQ-20 has a balanced 'overall agreement set', including both positively phrased items, agreement with which indicates psychological health, and negatively phrased items, agreement with which indicates psychological distress.

The items are scored on four-point response scales (Table I), ranging from 'better than usual' (score = 0) to 'much less than usual' (score = 3) (positively phrased items) or 'less than usual' (score = 0) to 'much more than usual' (score = 3) (negatively phrased items). The 20 single items are then summed (named Likert score), giving a possible scoring range from 0 to 60. An alternative scoring procedure is the GHQ score, which can be used for identifying 'cases'. The four response categories are treated as a binary response scale (scores 0 or 1), giving a possible scoring range of 20. With a cut-off point for 'case' identification between 3 and 4 in the GHQ-20, the sensitivity and specificity were 78% and 85% respectively compared with DSM-III criteria (Goldberg and Williams, 1988). This was used for estimation of the prevalence of 'true cases' in the present population (Table II).

The EORTC QLQ-C30 comprises six multi-term function scales measuring physical, role, social, emotional and cognitive function, and overall quality of life (Aaronson *et al.*, 1993). Three multi-item symptom scales measure pain, fatigue and emesis; six single items measure bowel function, breathing, appetite, sleeping disturbances and economic consequences of the disease. The emotional function scale and the single items were not included in the model for the multivariate analyses of the relationship between psychological distress and independent variables.

The 19-item head and neck cancer module, which has been developed at the NRH (Bjordal and Kaasa 1992), was designed to be used together with the EORTC QLQ-C30. The module is now being further developed along the guidelines laid down by the EORTC Study Group on Quality of Life (Sprangers *et al.*, 1993). The module included items assessing symptoms and side-effects especially relevant to head and neck cancer patients. In order to reduce the number of variables in the analyses, we tried to establish subscales within the module based on face validity, inter-item correlations, factor analyses and internal consistency in the present data set. This approach resulted in a three-item 'swal-

**Table I** Examples of scoring with the General Health Questionnaire (GHQ–20)

| | *Have you recently been able to concentrate on whatever you're doing?* | | | | |
|---|---|---|---|---|---|
| | *Better than usual* | *Same as usual* | *Less than usual* | *Much less than usual* | *Possible range* |
| Likert score | 0 | 1 | 2 | 3 | 0–60[a] |
| GHQ score[b] | 0 | 0 | 1 | 1 | 0–20[a,b] |

[a]High score, high level of psychological distress. [b]Cut-off point for case between 3 and 4.

**Table II** Calculation of estimated prevalence of 'true DSM-III cases'

$$\text{Estimated prevalence}^{a} = \frac{[\%\ \text{cases} - (100 - \text{specificity})]}{\dfrac{\text{sensitivity}}{100} - \dfrac{100 - \text{specificity}}{100}} = \frac{[31 - (100 - 85)]}{\dfrac{78}{100} - \dfrac{(100-85)}{100}} = 25.4$$

[a]Based on sensitivity = 78% and specificity = 85% (Goldberg and Williams, 1988).

lowing problem' scale which included items assessing problems with swallowing in general, trouble with swallowing bread and a tendency to swallow the wrong way. The factor loadings were 0.88, 0.75 and 0.43 for the three items respectively, and Cronbach's alpha for the scale was 0.83. No other scales were established.

All scales and single items in the EORTC QLQ-C30 and in the head and neck cancer module are scored on categorical scales, most of them with four categories (Table III). In accordance with the scoring instructions given by the EORTC Quality of Life Study Group, the scale scores are linearly transformed to 0–100 scores. A high score means a high level of functioning or a higher level of symptoms. In addition to the 'swallowing problem' scale, four of the items in the module with the highest mean scores in the entire group were included in the multivariate analyses: coughing, trouble with taste, dryness in the mouth and mucus production (mean scores 25, 21, 35 and 32 respectively).

### *Statistical analyses*

The statistical software SPSS PC+ version 4.0 was used in the statistical analyses. Differences in psychological distress between groups were tested with analysis of variance (ANOVA) (Likert score) or chi-square tests (GHQ score). The multivariate associations between psychological distress and the independent variables were tested both by stepwise multiple regression analyses (Likert score) and by stepwise logistic regression (GHQ score). Transformation (square root and log) or the Likert score in the multiple regression analyses did not change the results.

Twenty-three independent variables were tested; the 12 QOL variables in Table IV and 11 clinical/demographic variables: age, gender, level of education, living alone or not, randomisation (2 Gy × 5 *vs* 2.35 Gy × 4), disease site, disease stage, relapse (no/yes), secondary cancer (no/yes), the kind of surgery performed (none, minor, major), and whether the radiotherapy was given pre- or post-operatively. The QOL variables were treated as both continually and dichotomised response scales/items in the regression analyses with similar results. Results are reported for the dichotomised response scales/items (high/low – cut-off point at 50).

Because of the multiple testing, the statistical significance level of 0.01 was used in the analyses.

### Results

The level of psychological distress measured by the GHQ-20 was similar in both treatment groups (the CR group and the HR group). The mean Likert scores (s.d.) in the CR and HR groups were 20.8 (10.0) and 19.7 (8.3) respectively. The proportion of 'cases' (GHQ score>3) was 31% in the CR group and 32% in the HR group, giving an estimated calculated prevalence of 'true DSM-III cases' of 25% (Table II). Thus, in the subsequent univariate and multivariate analyses, the patients in the two groups were analysed together.

There were no statistically significant bivariate associations between psychological distress (the dependent variable) and independent sociodemographic or clinical variables such as age, gender, whether or not patients were living with spouse, level of education, diagnosis, stage of disease or treatment modality. Nor did recurrence after the randomised treatment ($n = 23$) or secondary primary cancers ($n = 19$) influence the level of psychological distress.

Patients reporting low physical, role, social or cognitive function or a high level of pain, fatigue or emesis (cut-off point 50 on the scales, named 'poor performance group') on the EORTC QLQ-C30 reported a higher level of psychological distress than patients with high levels of function or few symptoms (Table IV). Between 50% to 80% of patients in the 'poor performance group' were classified as 'cases' according to the GHQ score (>3). A similar strong association was found for patients reporting a high level of treatment-related side-effects (head and neck cancer module). Results are reported for the 'swallowing problem' scale and the four items with the highest mean scores (Table IV). Similar clinical and statistical significant associations ($P<0.01$) with GHQ Likert score were also found for patients with high level of other symptoms (score ≥ 50) in the head and neck cancer module, i.e. pain in the mouth, hoarseness, problems with talking on the telephone, dizziness and headache.

Stepwise multiple regression analyses were performed to explore the importance of the various QOL scales/single items and the clinical/demographic variables as predictors of psychological distress. The GHQ-20 Likert score was treated as the dependent variable, while the function/symptom scales in the EORTC QLQ-C30, the 'swallowing problem' scale and the symptoms items with highest mean score (coughing, trouble with taste, dryness in the mouth and mucus production) were treated as dichotomised independent variables (see Materials and methods section). In addition, 11 demographic/clinical variables were included as independent variables.

The cognitive and social function scales entered the model first, followed by pain (explaining 38% of the variance). Thereafter, stage II entered the model. These four variables accounted for 40% of the variance in psychological distress (Table V). Ninety-six patients (47%) had at least one of these four attributes. No other variables had a $P$-value between 0.001 and 0.05. The constant of 17.6 in Table V represents the calculated mean GHQ-20 Likert score in patients without any of the four attributes described in the table. The regression coefficients represent the increased/decreased score for each of the attributes in the model. Cognitive function, social function and stage II also entered the model in the logistic

**Table III** Examples of scoring with the EORTC QLQ-C30[a] and the head and neck cancer module

| | *Way of scoring* | | | | |
|---|---|---|---|---|---|
| *During the past week* | *Not at all* | *A little* | *Quite a bit* | *Very much* | *Possible range* |
| *EORTC QLQ-C30 function scales* | | | | | |
| Has your physical condition or medical treatment interfered with your social activities? | | | | | |
| 0–100 transformation | 100 | 66 | 33 | 0 | 0–100[b] |
| *EORTC QLQ-C30 symptom scales and the head and neck cancer module* | | | | | |
| Were you tired? | | | | | |
| Has your mouth been dry? | | | | | |
| 0–100 transformation | 0 | 33 | 66 | 100 | 0–100[c] |

[a]The European Organization for Research and Treatment of Cancer Core Quality of Life Questionnaire, 30-item version. [b]High score, high level of functioning. [c]High score, high level of symptoms.

Table IV Bivariate associations between psychological distress measured by GHQ-20 and patient-reported functions and symptoms in the EORTC QLQ-C30 and the head and neck cancer module

| *Independent variable* | | n | *Dependent variable GHQ-20 Likert score mean (s.d.)* | P* | *Number (%) of 'cases' GHQ score* | P** |
|---|---|---|---|---|---|---|
| *EORTC QLQ-C30, functioning scales*[a] | | | | | | |
| Physical function | High | 172 | 19.6 (9.0) | 0.025 | 47 (27) | 0.011 |
| | Low | 32 | 23.6 (9.7) | | 16 (50) | |
| Role function | High | 141 | 18.1 (7.6) | <0.001 | 30 (21) | <0.001 |
| | Low | 63 | 24.9 (10.7) | | 33 (52) | |
| Social function | High | 158 | 17.8 (6.9) | <0.001 | 30 (19) | <0.001 |
| | Low | 46 | 28.6 (11.1) | | 33 (72) | |
| Cognitive function | High | 175 | 18.3 (7.1) | <0.001 | 40 (23) | <0.001 |
| | Low | 29 | 31.7 (11.9) | | 23 (80) | |
| *EORTC QLQ-C30, symptom scales*[b] | | | | | | |
| Pain | Low level of symptoms | 175 | 18.7 (7.8) | <0.001 | 44 (25) | <0.001 |
| | High level of symptoms | 29 | 29.3 (11.7) | | 19 (66) | |
| Fatigue | Low level of symptoms | 164 | 18.3 (7.7) | <0.001 | 35 (21) | <0.001 |
| | High level of symptoms | 40 | 27.9 (10.9) | | 28 (70) | |
| Emesis | Low level of symptoms | 199 | 19.8 (8.45) | <0.001 | 59 (30) | 0.016 |
| | High level of symptoms | 5 | 39.0 (17.6) | | 4 (80) | |
| *Head and neck cancer module*[c] | | | | | | |
| Swallowing problem scale | Low level of symptoms | 172 | 19.0 (8.1) | 0.001 | 43 (25) | <0.001 |
| | High level of symptoms | 28 | 27.3 (11.7) | | 18 (64) | |
| Coughing | Low level of symptoms | 172 | 19.3 (8.6) | <0.001 | 44 (26) | <0.001 |
| | High level of symptoms | 30 | 25.5 (10.1) | | 18 (60) | |
| Trouble with taste | Low level of symptoms | 173 | 19.6 (8.8) | 0.025 | 47 (27) | 0.004 |
| | High level of symptoms | 30 | 23.7 (10.9) | | 16 (53) | |
| Dryness in the mouth | Low level of symptoms | 146 | 19.2 (9.1) | 0.009 | 36 (25) | 0.002 |
| | High level of symptoms | 58 | 22.9 (9.0) | | 27 (47) | |
| Mucus production | Low level of symptoms | 144 | 19.3 (9.2) | 0.019 | 35 (24) | <0.001 |
| | High level of symptoms | 58 | 22.7 (8.9) | | 28 (48) | |

**P*-value (ANOVA). ***P*-value (chi-square). [a]High function = mean scale score > 50. Low function = mean scale score ⩽ 50. [b]Low level of symptoms = mean scale score <50. High level of symptoms = mean scale score ⩾ 50. [c]Low level of symptoms = mean scale or single item score <50. High level of symptoms = mean scale or single item score ⩾ 50. *n* varies because of missing items.

regression analysis. In addition a high level of coughing also entered the model.

### Discussion

The level of psychological distress according to the GHQ-20 (31% of 'cases', 25% satisfying DSM-III criteria, Table II) was higher than anticipated, on the basis of clinical experience, in the present cross-sectional study of head and neck cancer patients. Considering that these patients are long-term survivors, 7–11 years after treatment, these findings are worrying. The patients have completed the clinical follow-up programme (which seldom focus on these problems), and they are not being offered systematic support or psychological treatment.

A somewhat higher prevalence of depression (40% case scores in the Geriatric Mental State Schedule) than in this study was found in a small pilot study of 48 male patients with buccopharyngeal cancer (age > 60 years) (Morton *et al.*, 1984). However, these findings can be explained by the shorter follow-up time (no evidence of disease for at least 6 months), and by the fact that six of the patients had their larynx removed and 17 of them had receive salvage surgery after failed radiotherapy. Forty-seven per cent of the patients receiving salvage surgery were classified as depressed.

To our knowledge, there are no current data on GHQ-20 in a normal population. A lower proportion of 'case scores' compared with our population was found in male patients in the age group 55–74 in a population survey in Great Britain (25% of 'cases', GHQ-30) (Cox *et al.*, 1987). A similar level of 'cases' in GHQ has been found in patients in general practice (33%, GHQ-30) (Cleary *et al.*, 1982). However,

Table V Stepwise multiple regression analysis with GHQ-20 Likert score as the dependent variable

| *Independent variable*[a] | *Regression coefficient* | *Standard error* | P-*value* |
|---|---|---|---|
| Low cognitive function[b] | 8.6 | 1.6 | <0.001 |
| Low social function[b] | 5.5 | 1.4 | <0.001 |
| High level of pain[b] | 5.7 | 1.6 | <0.001 |
| Stage II[c] | −3.5 | 1.2 | 0.006 |
| Constant | 17.6 | 0.6 | |
| $R^2 = 0.40$ | | | |

[a]Independent variables in the analysis: 12 QOL variables and 11 clinical/demographic variables. [b]Cut-off point = 50. [c]In the variance analysis (ANOVA), patients in stages I, II, III and IV had mean Likert scores of 20.9, 17.1, 20.6 and 21.6 respectively ($P = 0.117$).

patients with psychological distress have an increased tendency to seek medical attention (Finlay-Jones and Burvill, 1978). Groups of cancer patients similar to those in our study are difficult to find owing to the very special composition with regard to background variables in the latter population. In a study using the Brief Symptom Inventory, 22% of 273 Hodgkin's disease survivors had a score above the cut-off point for psychiatric diagnosis (Kornblith *et al.*, 1992). In another study of the long-term effects of cancer treatment, young adult cancer patients (50% with Hodgkin's disease) had similar scores to their neighbours in most areas of subjective well-being, including a low level of anxiety and depression measured by the Hospital Anxiety and Depression (HAD) Scale (frequency of cases not specified) (Olweny *et al.*, 1993). A high level of psychological distress was found in a Norwegian study of cancer patients who had received palliative radiotherapy; 69% had a 'case' score on GHQ-20, and the mean Likert score was 27.3 (Kaasa *et al.*, 1993).

The variety of methods of assessment that have been used in studies of psychological distress, anxiety and depression in cancer patients makes it difficult to compare the findings. Furthermore, the study populations have been selected in various ways from patient populations according to different criteria. Thus, there seems to be an urgent need for a standardised method of measuring psychological distress (anxiety/depression) in cancer patients. In Norway, we are trying to limit the use of such questionnaires to the GHQ-20 and the HAD scale in clinical cancer trials. The latter scale is also being used as a standard instrument for measuring psychological distress by the Medical Research Campaign (MRC) in the UK.

Psychological support for cancer patients has in general low priority, probably because of the focus on tumour control and other physical aspects of the disease in clinical trials and examinations after treatment. Even less attention has been paid to psychological factors in cured patients.

And yet it is important to identify patients with such problems. The most obvious way of doing this is by interviews at clinical follow-ups. However, patients suffering from mood disorders seldom complain spontaneously about their difficulties, unless the physician poses the appropriate questions and individual clinicians differ in their ability to detect psychological disorders (Goldberg, 1984). Moreover, the patients may not expect the physician to enquire into psychological areas in check-ups. Both patients and staff could be under the impression that depression and anxiety are inevitable consequences of having cancer. For these reasons psychological distress and other symptoms in these patients are probably under-reported or not diagnosed (Maguire *et al.*, 1980).

This under-reporting problem could be dealt with in several ways. One is to include these aspects in the training of medical students, in connection with the curative biological end point. Specific training in communication with patients with life-threatening or chronic diseases is needed (Razavi *et al.*, 1990), and should be mandatory for all physicians dealing with cancer patients.

The use of structured interviews and validated questionnaires facilitates the detection of patients with psychological distress. The structured psychiatric interview is the best means we have for diagnosing psychological morbidity, but structured interviews are time-consuming and often require special training. A validated questionnaire such as the GHQ-20 can be used to alert clinicians to the possibility of psychological disorder in their patients. When this is used in a clinical setting, and a patient is found to have a 'case' score, the clinician could follow up on the particular symptoms indicated by the patient. A two-step screening process like this is likely to identify most of the patients with a true-positive 'case' score.

Increased knowledge of the predictors of psychological distress may help clinicians to identify patients at risk. In the present study, psychological distress was not significantly associated with the randomised treatment, which is what we expected to find. In the univariate analyses, we found a strong association between psychological distress and the various QOL variables, including most of the items in the head and neck cancer module. However, reduced cognitive and social function and pain were the only significant QOL variables in the regression model. It is important to note that these three variables together explained 38% of the variance in psychological distress. The reason for the negative association with stage II alone is difficult to explain. Neither the sociodemographic variables such as age, gender and education nor the other variables reflecting disease and treatment were significantly associated with psychological distress as measured by GHQ-20 in this population. Thus, the identification of pain and/or reduced cognitive and social function in cured patients should alert the physician to the possible necessity for treatment, including programme intervention.

Being limited by its cross-sectional design, the present study could not be used to identify various QOL scales/single items as prognostic factors for psychological distress. We do not know whether patients with high levels of psychological distress report symptoms more readily or whether high levels of symptoms cause psychological distress. However, in the study of the validity of the present QOL questionnaire in head and neck cancer patients, the level of the different self-reported symptoms reflected the expected acute, subacute and late toxicity of treatment (Bjordal and Kaasa, 1992). A prospective longitudinal study of quality of life in head and neck cancer patients, with special focus on psychological distress, has been initiated in Norway and Sweden. It may provide valid data concerning fluctuations in psychological distress over time and prognostic factors for psychological distress during and after treatment.

What can we offer patients identified as suffering from psychological distress or patients at risk? In a study of patients undergoing cancer treatment, the large majority of those (45%) with a psychiatric diagnosis had highly treatable disorders (Derogatis *et al.*, 1983). However, most patients identified as having psychological distress will not require specialist referral, but can be treated in a cost-effective way by providing social support or medication. All patients are likely to benefit from optimal communication and support by the clinician and the follow-up team, or at the primary health care level.

In the present study, a high prevalence of psychological distress was found in long-term survivors of head and neck cancer, especially in patients with impaired cognitive or social function or with pain. Clinicians need to be aware of this morbidity, and their ability to detect patients with psychological disorders needs to be improved. One way of doing this would be to include specific training in communication in the medical studies. We also need more information about prognostic factors for psychological distress, in order to be able to prevent these problems. The GHQ-20 can facilitate this process in a clinical setting. If these patients are identified, psychological distress can be reduced and treated, and the patients might be able to cope more effectively, which may improve their quality of life.

**Acknowledgement**

The authors wish to thank A Mastekaasa for valuable comments and help with the statistical analyses. We thank H Vermund for initiating the randomised study and J Tausjø and other clinicians at the Norwegian Radium Hospital for entering patients in the randomised study. We also thank JF Evensen for his support, H Høst for comments on the manuscript and B Moldaunet and M Birkeland Midthus for invaluable help with data processing and the mailing of letters to the patients. The study was supported by Grant No. 88283/002 from the Norwegian Cancer Society.

### References

AARONSON NK, AHMEDZAI S, BERGMAN B, BULLINGER M, CULL A, DUEZ N, FILIBERTI A, FLECHTNER H, FLEISHMAN SB, DE HAES JCJM, KAASA S, KLEE M, OSOBA D, RAZAVI D, ROFE PBC, SCHRAUB S, SNEEUW K, SULLIVAN M AND TAKEDA F. FOR THE EUROPEAN ORGANIZATION FOR RESEARCH AND TREATMENT OF CANCER, STUDY GROUP ON QUALITY OF LIFE. (1993). The EORTC QLQ-C30: A qualify of life instrument for use in international clinical trials in oncology. *J. Natl. Cancer Inst.*, **85**, 365–376.

AMERICAN PSYCHIATRIC ASSOCIATION. (1980). *Diagnostic and Statistical Manual of Mental Disorders*, 3rd edn (DSM-III). American Psychiatric Association Press: Washington, DC.

BJORDAL K AND KAASA S. (1992). Psychometric validation of the EORTC core quality of life questionnaire, 30-item version and a diagnosis-specific module for head and neck cancer patients. *Acta Oncol.*, **31**, 311–321.

BJORDAL K, KAASA S AND MASTEKAASA A. (1994). Quality of life in patients treated for head and neck cancer. A follow-up study seven to 11 years after radiotherapy. *Int. J. Radiat. Oncol. Biol. Phys.*, **28**, 847–856.

BOLUND C. (1985). Suicide and cancer: II. Medical and care factors in suicides by cancer patients in Sweden, 1973–1976. *J. Psychosocial Oncol.*, **3**, 31–52.

BREIBART W AND HOLLAND J. (1988). Psychosocial aspects of head and neck cancer. *Semin Oncol.*, **15**, 61–69.

CLEARY PD, GOLDBERG ID, KESSLER LG AND NYCZ GR. (1982). Screening for mental disorders among primary care patients. *Arch. Gen. Psychiatry*, **39**, 837–840.

COX B, BLAXTER M, BUCKLE A, FENNER NP, GOLDING J, GORE M, HUPPERT F, NICKSON J, ROTH M, STARK J, WADSWORTH M AND WICHELOW M. (1987). *The Health and Lifestyle Survey*. Health Promotion Research Trust: Cambridge.

CRAIG TJ, COMSTOCK GW AND GEISER PB. (1974). The quality of survival in breast cancer: a case-control comparison. *Cancer*, **33**, 1451–1457.

DAVID DJ AND BARRITT JA. (1977). Psychosocial aspects of head and neck cancer surgery. *Aust. NZ. J. Surg.*, **47**, 584–589.

DAVID DJ AND BARRITT JA. (1982). Psychosocial implications of surgery for head and neck cancer. *Clin. Plastic Surg.*, **9**, 327–336.

DEROGATIS LR, MORROW GR, FETTING J, PENMAN D, PIASETSKY S, SCHMALE AM, HENRICHS M AND CARNICKE CLM. (1983). The prevalence of psychiatric disorders among cancer patients. *JAMA*, **249**, 751–757.

DRETTNER B AND AHLBOM, A. (1983). Quality of life and state of health for patients with cancer in the head and neck. *Acta Otolaryngol.*, **96**, 307–314.

DROPKIN MJ. (1989). Coping with disfigurement and dysfunction after head and neck cancer surgery: a conceptual framework. *Semin. Oncol. Nurs.*, **5**, 213–219.

FINLAY-JONES RA AND BURVILL PW. (1978). Contrasting demographic patterns of minor psychiatric morbidity in general practice and the community. *Psychol. Med.*, **8**, 455–466.

FOBAIR P, HOPPE RT, BLOOM J, COX R, VARGHESE A AND SPIEGEL D. (1986). Psychosocial problems among survivors of Hodgkin's disease. *J. Clin. Oncol.*, **4**, 805–814.

GOLDBERG DP. (1984). The recognition of psychiatric illness by non-psychiatrists. *Aust. NZ J. Psychiatr.*, **18**, 128–134.

GOLDBERG D AND WILLIAMS P. (1988). *A User's Guide to the General Health Questionnaire*. NFER-Nelson: Windsor, Berkshire.

GOTAY CC AND MOORE TD. (1992). Assessing quality of life in head and neck cancer (review). *Qual. Life Res.*, **1**, 5–17.

HOLMEN J, MIDTHJELL K, BJARTVEIT K, HJORT PF, LUNDLARSEN PG, MOUM T, NÆSS S AND WAALER HT. (1990). The North-Trøndelag health survey 1984–1986. Purpose, background and methods. Participation, non-participation and frequency distributions, Report No. 4. National Institute of Public Health, Unit for Health Services Research: Oslo and National Institute of Public Health, Community Medicine Research Centre: Verdal, Norway.

HUGES JE. (1987). Psychological and social consequences of cancer. *Cancer Surv.*, **1**, 455–475.

KAASA S, AASS N, MASTEKAASA A, LUND E AND FOSSÅ SD. (1991). Psychosocial well-being in testicular cancer patients. *Eur. J. Cancer*, **27**, 1091–1095.

KAASA S, MALT U, HAGEN S, WIST E, MOUM T AND KVIKSTAD A. (1993). Psychological distress in cancer patients with advanced disease. *Radiother. Oncol.*, **27**, 193–197.

KORNBLITH AB, ANDERSON J, CELLA DF, TROSS S, ZUCKERMAN E, CHERIN E, HENDERSON E, WIESS RB, COOPER MR, SILVER RT, LEONE L, CANNELLOS GP, GOTTLIEB A AND HOLLAND JC. (1992). Hodgkin disease survivors at increased risk for problems in psychosocial adaption. *Cancer*, **70**, 2214–2224.

MAGUIRE GP, TAIT A, BROOKE M, THOMAS C, HOWAT JMT, SELLWOOD RA AND BUSH H. (1980). Psychiatric morbidity and physical toxicity associated with adjuvant chemotherapy after mastectomy. *Br. Med. J.*, **281**, 1179–1180.

MORTON RP, DAVIES ADM, BAKER J, BAKER GA AND STELL PM. (1984). Quality of life in treated head and neck cancer patients: a preliminary report. *Clin. Otolaryngol.*, **9**, 181–185.

OLSON M AND SHEDD DP. (1978). Disability and rehabilitation in head and neck cancer patients after treatment. *Head Neck Surg.*, **1**, 52–58.

OLWENY CLM, JUTTNER CA, ROFE P, BARROW G, ESTERMAN A, WALTHAM R, ABDI E, CHESTERMAN H, SESHADRI R, SAGE E, ANDARY C, KATSIKITIS M, ROBERTS M AND SELVANAYAGAM S. (1993). Long-term effects of cancer treatment and consequences of cure. Cancer survivors enjoy quality of life similar to their neighbours. *Eur. J. Cancer*, **29A** (6), 826–830.

RAZAVI D, DELVAUX N, FARVACQUES C AND ROBAYE E. (1990). Screening for adjustment disorders and major depressive disorders in cancer in-patients. *Br. J. Psychiatr.*, **156**, 79–83.

SPRANGERS MAG, CULL A, BJORDAL K, GROENVOLD M AND AARONSON NK FOR THE EORTC STUDY GROUP ON QUALITY OF LIFE. (1993). The European Organization for Research and Treatment of Cancer approach to quality of life assessment: Guidelines for developing questionnaire modules. *Qual Life Res.*, **2**, 287–295.

TURESSON I AND NOTTER G. (1984). The influence of fraction size in radiotherapy on the late normal tissue reaction: II. Comparison of the effects of daily and twice-a-week fractionation on human skin. *Int. J. Radiat. Oncol. Biol. Phys.*, **10**, 599–606.

VAN DONGEN-MELMAN JEWM AND SANDERS-WOUDSTRA JAR. (1986). Psychosocial aspects of childhood cancers: a review of the literature. *J. Child. Psychol. Psychiatr.*, **27**, 145–180.